# The attitudes of psychiatric patients towards COVID-19 vaccination in China: a cross-sectional study

**DOI:** 10.1186/s12888-021-03484-9

**Published:** 2021-09-29

**Authors:** Xin Ren, Fang Shen, Yan Gui, Weixin Wang, Baoping Xing, Wanli Huang

**Affiliations:** Tongde Hospital of Zhejiang Province, Mental Health Center of Zhejiang Province, No. 234 Gucui Road, Xihu District, Hangzhou, Zhejiang Province China

**Keywords:** Psychiatric patient, Family caregiver, Attitudes, COVID-19, Vaccination

## Abstract

**Background:**

The COVID-19 pandemic has become a global health catastrophe. By far, there has been no specific antiviral treatment for COVID-19. Developing a vaccine against COVID-19 appeared to be the most cost-effective strategy to stop the repeated outbreak. This study aimed to investigate the attitudes of psychiatric patients with regards to COVID-19 vaccination and potential factors that might influence their decision-making process.

**Methods:**

Psychiatric patients participated in this cross-sectional survey in China. Family caregivers, usually a guardian or next of kin completed the questionnaire when the patient is unable to consent. Data was collected via an online self-administered questionnaire. This questionnaire focused on four main attributes: (1) sociology-demographic characteristics, such as age and sex; (2) questions relating to the COVID-19 pandemic, such as perceived risk of COVID-19; (3) Flu vaccination history; and (4) attitude towards COVID-19 vaccination and affected factors, such as preferred vaccine type and vaccination site. The associated factors that influenced vaccination acceptance were analyzed by Chi-square analysis and binary logistic regression.

**Results:**

In total, 416 individuals were recruited, from which 408: 229 patients and 179 family caregivers completed the online survey (response rate: 98.1%). 78.7% of the participants (178 patients and 143 family caregivers) said they intended to receive vaccination once the COVID-19 vaccine became available on the market. Our results showed that participants would have a greater likelihood of joining the COVID-9 immunization programme if the people they knew (community residents or their friends and relatives) presented with high vaccine coverage (OR = 0.24; 95% CI:0.09–0.59). If the pandemic returns, participants were also more likely to accept vaccination (OR = 0.21; 95% CI:0.07–0.62). Moreover, those who believed that the vaccination was an important way to control the COVID-19 pandemic also showed a tendency to receive vaccination (OR = 0.21; 95% CI:0.11–0.40). For those who did not intend to get vaccinated either for themselves or their psychiatric family member, the safety of vaccine was their main concern (71.3%).

**Conclusions:**

This study showed a high acceptance rate for COVID-19 vaccination amongst psychiatric patients, while worries about the safety of vaccine led to refusal towards vaccination. To increase vaccination uptake amongst this vulnerable group, the public health messaging should include updated vaccination coverage in local communities, and the number of newly COVID-19 infected cases. Specific information about vaccine safety concerning psychiatric patients; as well as the importance of vaccination in controlling the pandemic should be explained in detail.

**Supplementary Information:**

The online version contains supplementary material available at 10.1186/s12888-021-03484-9.

## Background

The novel severe acute respiratory syndrome coronavirus 2 (SARS-CoV-2; COVID-19) emerged in December 2019. The COVID-19 pandemic has become a global health catastrophe, causing over 120 million confirmed cases and 2 million deaths worldwide [1].

Although many countries imposed strict restrictions to contain the virus, serious challenges still remained with regards to its highly infectious characteristics. Early in the pandemic, no clinical approved effective drugs was available [2]. Non-pharmaceutical interventions (e.g.. social distancing) were effective [3], however, implementing those interventions for a long term would cause damaging sociological [4] and psychological consequences [5]. Thus, developing a vaccine against COVID-19 appeared to be the most effective strategy to stop repeated outbreak [6].

National institutions and vaccine companies responded rapidly to this critical need. On 31st December 2020, the World Health Organization (WHO) approved a COVID-19 vaccine from Pfizer/BioTech for emergency use [7], and several other candidates were undergoing different phases of clinical trials [8]. Despite the scientific community and related industries working hard to create a vaccine against COVID-19, the mutations of the coronavirus genome might present difficulties with vaccine development [9], and the efficacy and safety of the vaccine remained a concern [10].

Although vaccines have been approved for emergency use in certain high risk groups [11], and a lot of efforts were made to improve their efficacy, the roll out of mass vaccination against COVID-19 would be complicated. To ensure a successful implementation of vaccine, our government planned to prioritize recipient groups and provide the COVID-19 vaccines for free. Also, national-level strategies have been made to guide and navigate through the vaccine roll-out process. While other nations are still combating the virus, China is now recovering; this might lead to public presumption that vaccination was unnecessary. In China, there are routinely offered vaccines/Category 1 free of charge to every child under the age of 14. While all of these vaccines are free, there are other vaccines/Category 2 vaccines for children and adults that are self-funded. Category 2 vaccines such as flu vaccine are taken up according to self-risk assessment. Self-perceived low risk of certain infectious diseases attributes to low vaccination adherence [12, 13], which explained why some optional and self-paid vaccines, still presented with deficient coverage. For example, only 10.5% of Chinese adults have been vaccinated against influenza [14]; this compared to 37 to 95% of adults in the United States [15]. Moreover, past incidents of expired or improperly stored vaccines have driven fervent debate amongst the public [16]. These factors also contributed to hesitation towards vaccination, especially in certain vulnerable groups, such as psychiatric patients. It has been established that during the pandemic, psychiatric patients have been associated with an increased risk of COVID-19 mortality and severity [17, 18]. Vaccination for COVID-19 is of paramount importance, and published studies urged governments to prioritize vaccination for psychiatric patients [19–22]. It is critical to evaluate the vaccination intention amongst this vulnerable group. Thus, in the present study, we aimed to investigate the attitudes of psychiatric patients with regards to COVID-19 vaccination and the potential factors that might influence acceptance for vaccination. These insights would offer empirical evidence to increase vaccination uptake and help the government to generate appropriate vaccination strategies.

## Methods

### Study design and sampling

We conducted an online cross-sectional survey using WeChat combined with SurveyStar (equivalent to SurveyMonkey) in Tongde hospital of Zhejiang Province, Hangzhou, China. The targeted participants were psychiatric patients. Participants were approached by the research team and recruited in waiting areas, patient rooms, and inpatient units. Eligible patients were aged over 18, able to understand and complete the survey, and had been previously diagnosed with schizophrenia, bipolar disorder, major depression disorder, generalized anxiety disorder or other mental disorders, according to the International Classification of Disease-10 (ICD-10). Patients were encouraged to complete the questionnaire themselves where possible. Family caregivers, usually a guardian or next of kin, would be recruited to complete the questionnaire when the patient is unable to consent. Under this circumstance, the patient did not need to be recruited. The family caregivers needed to meet the same first two criteria as the psychiatric patient and be responsible for taking care of the patients. This study was approved by Tongde Hospital of Zhejiang Province Ethics Board (Reference number: XMSC20210023).

### Data collection and measures

Participants used WeChat (a popular social media application in China) to scan a Quick respond (QR) code, which is a type of barcode that can be read by a digital device and stores information as a series of pixels in a square-shape grid, displayed on the research team’s digital device (a smartphone or a tablet). Then the participants would be directed to a survey page on SurveyStar. For those who did not have a smartphone or a WeChat account, the survey would be completed on the research team’s WeChat account on the tablet. By consenting to complete our questionnaires, they are agreed to participate in this study. Data was obtained between 9th January 2021 and 9th February 2021.

The research team conducted a comprehensive literature review on MEDLINE, the Cochran’s Library, Embase, Web of Science and Chinese databases such as China national knowledge infrastructure to explore essential factors that might affect the preferences and attitude of the patients when making decisions related to vaccination uptake. Two researchers completed the literature review, and all team members discussed the findings. Based on the findings of previous research, we designed a self-administered questionnaire. Then, we randomly chose two patients and three family caregivers to test the questionnaire. According to their feedback, we refined some questions, and adjusted their expression, to ensure clarity. The final version of the questionnaire (See Table S1) focused on four main attributes: (1) socio-demographic characteristics, such as age, sex, marital status, education, employment status, and income; (2) questions relating to the COVID-19 pandemic, such as perceived risk of the COVID-19; (3) Flu vaccination history; and (4) attitude towards COVID-19 vaccination and factors that influenced decision-making process, such as vaccine fees, convenience and healthcare professional recommendations. The questionnaire was presented to all participants in Chinese to assure accurate response. There were 31 questions in total and it would take participants about three to 5 min to complete. Some questions were single-choice questions; others required yes/no responses. The last question was optional and open-ended with a blank space. It was a question to encourage participants to share their concern over COVID-19 vaccination. Since the open-ended question was not an answer to a specific question, this data would not be analyzed..

### Statistical analysis

The primary outcome of the study was the acceptance of the COVID-19 vaccination. All participants were divided into two groups, depending on the answer to the question: ‘Would you like to be vaccinated if COVID-19 vaccines become available’. Participants who chose ‘yes’ would be classified into the vaccine-accept group. Those who selected ‘no’ were classified into the vaccine-refuse group. All analyses were conducted using the Statistical Package for the Social Sciences (SPSS) 22.0 (IBM Crop., Armonk, New York, the United States). Frequency analysis was adopted to describe all variables with regard to the distribution of features. Baseline characteristics between the vaccine-accept and vaccine-refuse groups were first investigated by the Chi-squared test to identify the significant factors amongst the variables to ensure reliable results. Significant factors at the 5% level of the Chi-square analysis were then included in binary logistic regression, which was subsequently performed between the two groups (vaccine-accept group and vaccine-refuse group) to identify the specific factors that influenced vaccination acceptance.

## Results

### General characteristics of the participants

In total, 416 individuals were recruited, from which 408: 229 patients and 179 family caregivers completed the online survey (response rate: 98.1%). Eight patients who did not consent or complete the questionnaire were excluded. A higher proportion of participants were female (63.2%) and between 18 and 44 years (66.4%). Most participants were married (55.9%) or lived with their parents (30.6%). More than half held a bachelor’s degree (59.6%) or reported that they were employed as full time (51.9%). Regarding personal income, approximately 73.3% had an annual salary of over 60,000 Chinese Yuan. A large number of patients were diagnosed with major depressive disorder (32.8%), followed by bipolar disorder (27.5%), generalized anxiety disorders (17.4%), other psychotic disorder (12.7%) and schizophrenia (9.6%). Most participants (81.4%) stated they lived in urban areas, and 49.7% of the subjects perceived their health status as being fair.

Only 13.0% of the participants (18 patients and 35 family caregivers) perceived themselves or their family member as high risk of being infected. Although they expressed that the pandemic hampered their work or daily life (33.6%), few subjects perceived that their income had been significantly affected (21.3%). Furthermore, a relatively low proportion of participants had been vaccinated against influenza previously (21.6%). More detailed information of the participants is provided in Table 1.

**Table 1:** The basic characteristics, risk perception, impact of COVID-19 and Flu vaccination history of the participants

Items	Participants (***n*** = 408)	n (%)
Identity		
Patient	229	56.1
Family caregiver	179	43.9
Diagnosis		
Major Depression Disorder	134	32.8
Bipolar Disorder	112	27.5
Generalized Anxiety Disorder	71	17.4
Others	52	12.7
Schizophrenia	39	9.6
Sex		
Female	258	63.2
Male	150	36.8
Age group		
18–44	271	66.4
45–59	111	27.2
60 and above	26	6.4
Marriage status		
Married	228	55.9
Unmarried	161	39.5
Others (divorced, widowed)	19	4.6
Highest level of education		
Bachelor and above	243	59.6
Middle or high school	158	38.7
Primary school and below	7	1.7
Working status		
Full-time employed	212	51.9
Students	65	15.9
Part-time employed	55	13.5
Retired	46	11.3
Unemployed	30	7.4
Region		
Urban	332	81.4
Rural	76	18.6
Living status		
With parents	125	30.6
With children	123	30.2
Independent or with partners	109	26.7
With parents and children	51	12.5
Annual personal income		
Over 60,000 (¥)	299	73.3
Less or equal to 60,000 (¥)	109	26.7
How do you think of your health status?		
Fair	203	49.7
Good	181	44.4
Poor	24	5.9
How do you perceived your risk of getting COVID-19 infected		
Low or very low	182	44.6
Fair	173	42.4
High or very high	53	13.0
Did the pandemic affect your daily life or work?		
Fair	180	44.1
Large or very large	137	33.6
Small or very small	91	22.3
Did the pandemic affect your income?		
Fair	164	40.2
Small or very small	157	38.5
Large or very large	87	21.3
Did you receive Flu vaccination in past seasons?		
No	320	78.4
Yes	88	21.6

### Attitude and influencing factors of the COVID-19 vaccine

Many participants (86.8%) reported they were aware that certain groups of people had already received the COVID-19 vaccination. The predominant means of gaining information about the COVID-19 vaccine were from mass media (39.5%) and social media (42.1%); authorities, such as health departments, were not used often at providing information. Approximately half of the participants rated themselves as ‘roughly clear’ when questioned ‘which best describe your understanding about COVID-19 vaccines’. With regards to the vaccine’s features, 43.6% of subjects attached more importance to its efficacy in comparison to other two features of the vaccines, safety and protection duration.

Three hundred and twenty-one participants (78.7%) said they intended to get vaccinated or would agree to vaccinate for who they are cared for once the COVID-19 vaccine was available on the market. Furthermore, 64.5% said they would recommend others to take the vaccine. Amongst the participants who rejected the vaccine, their main concern was potential adverse effects. Participants regarded the advice from healthcare professionals and government favourably. Analysis showed that 63.0% of subjects expressed that they were more likely to vaccinate against COVID-19 if doctors or disease control personnel suggested this option to them. Furthermore, 88.2% of subjects considered that they would have a greater likelihood of joining the COVID-19 vaccination program, if more people they knew (community residents or friends and relatives) had been vaccinated against COVID-19. They also agreed that they would be more likely to receive a vaccine if that helped to protect their children and the elderly (90.0%). The belief of possible resurgence of the pandemic in future was shown to the raised likelihood of getting vaccination in our subjects (89.2%).

Our participants favoured hospitals (57.6%) as vaccination site more than community health centre or health screen centre. Our results demonstrated that they preferred vaccines that were funded entirely by the government. More participants selected the Chinese vaccines and agreed that the vaccine was crucial to contain the virus. Nevertheless, negative news, such as case reports of adverse effect related to the COVID-19 vaccine appeared to lower their possibility of accepting the vaccination (63.7% of subjects). More detailed information of the participants’ attitude and preference towards COVID-19 vaccination is provided in Table 2.

**Table 2:** The attitude and influencing factors of COVID-19 vaccination amongst the participants

Items	Participants (n = 408)	n (%)
Which best describe your understanding about COVID-19 vaccines?		
Roughly clear	228	55.9
Not clear at all	133	32.6
Very clear	47	11.5
Do you know anyone who might have been vaccinated against COVID-19?		
Yes	354	86.8
No	54	13.2
Where do you get information about COVID-19 vaccines from most often?		
Social media	172	42.1
Mass media	161	39.5
Authorities	53	13.0
Others	22	5.4
What concern you the most about COVID-19 vaccines?		
Effectiveness	178	43.6
Safety	140	34.3
Protection duration	90	22.1
Would you like to be vaccinated if COVID-19 vaccines become available?		
Yes	321	78.7
No	87	21.3
What is the reason for rejecting vaccination? (Only assess for vaccine refuse group, *n* = 87)		
Worry about the safety	62	71.3
Worry about the mutation	22	25.3
Worry about the cost	3	3.4
Will you encourage others to get vaccinated?		
Yes	263	64.5
No	145	35.5
Which of the following people’s suggestions would increase your likelihood of getting vaccinated?		
Authority or Doctors	257	63.0
Family	119	29.2
Friends	32	7.8
You are more likely to have vaccines if there is high vaccine coverage amongst community residents or relatives and friends.		
Yes	360 48	88.2 11.8
No		
You are more likely to have vaccines if the pandemic returns.		
Yes	364	89.2
No	44	10.8
You are more likely to have vaccines in order to protect children or the elderly in your family.		
Yes	367	90.0
No	41	10.0
Which place do you think is most suitable for vaccination?		
Hospitals	235	57.6
Community health centre	158	38.7
Health screen centre	15	3.7
How do you think vaccines should be charged?		
Free	231	56.6
Partially paid by individual	169	41.4
All paid by individual	8	2.0
Which vaccine would you prefer to be vaccinated?		
Chinese vaccines	340	83.3
Imported	68	16.7
You are less likely to have vaccines if negative news reported against COVID-19 vaccines.		
Yes	260	63.7
No	148	36.3
Do you think the COVID-19 vaccine is crucial to control the pandemic?		
Yes	325	79.7
Not sure	74	18.1
No	9	2.2

Here we presented some opinions left in the open-ended question. It was apparent that they seemed to worry about the side effects of the vaccines and whether vaccines were safe for a specific group of people; Some responses include:

*‘I have taken medications to control the symptoms of General Anxiety Disorder for three years, and my question is whether receiving vaccination would affect the current treatment I am under?’ (Survey participant #32, patient)*

*‘The Safety of the vaccine should take priority over any other matter. Since our country has a large population, it would be a disaster if any serious vaccine related problems come out.’ (Survey participant #283, patient)*

Furthermore, they voiced their suggestions for vaccine campaigns as follows.

*‘I heard that certain groups of people are not suitable to receive this vaccine, I hope the government gives us a clear and well-organized instruction when implementing a immunity program.’ (Survey participant #108, family caregiver)*

*‘I’m very pro-vaccines and want to vaccinate against COVID-19 as soon as possible, but I really don’t know how we can apply or where to get the vaccine.’ (Survey participant #70, patient)*

### Influencing factors of vaccination acceptance

Overall, 78.7% of our participants stated that they intended to get vaccinated or would agree to vaccinate for who they were cared for. The results of the Chi-square tests are shown in Table S2. Overall, the intention to be vaccinated was significantly (*p* < 0.05) influenced by sex, age, perceived risk of COVID-19, high vaccine coverage of surrounding people, possible return of pandemic, recommending vaccination to others, the belief of the vaccination would protect their children or the elderly, and the perception that vaccination was the key to keep COVID-19 under control. We used binary logistic regression between the two groups to identify the factors that might influence their decision to undergo vaccination. Table 3 presents the result of regression. This analysis showed that if most of a subject’s community residents or relatives and friends were vaccinated (odds ratio [OR] = 0.24; 95% confidence interval [95% CI]:0.09–0.59) or if the pandemic returns (OR = 0.21; 95% CI:0.07–0.62), participants were more likely to accept vaccination. Moreover, those who believed that the vaccination was a significant way to control the COVID-19 pandemic also showed a tendency to receive vaccination (OR = 0.21; 95% CI:0.11–0.40).

**Table 3:** Influencing factors on vaccination acceptance between the vaccine-accept group and vaccine-refuse group

Items	OR	SE	*p*-Value	95% C.I.
Sex				
Male	0.76	0.34	0.414	0.39–1.47
Female	Ref			
Age group				
18–44	1.06	0.61	0.921	0.32–3.50
45–59	0.60	0.68	0.459	0.16–2.29
60 and above	Ref			
Perceived risk of infection				
High or very high	0.67	0.52	0.440	0.24–1.84
Fair	0.60	0.33	0.116	0.31–1.14
Low or very low	Ref			
More likely to vaccinate if communities presented with high vaccine coverage or if most of their relatives and friends had been vaccinated				
Yes	0.24	0.47	0.002	0.09–0.59
No	Ref			
More likely to vaccinate if the pandemic returns				
Yes	0.21	0.55	0.005	0.07–0.62
No	Ref			
More likely to vaccinate in order to protect children or the elderly in family				
Yes	0.50	0.55	0.212	0.17–1.48
No	Ref			
Less likely to vaccinate if negative news about the COVID-19 vaccine come out				
Yes	1.85	0.35	0.078	0.93–3.67
No	Ref			
The COVID-19 vaccine is crucial to control the pandemic.				
Yes	0.21	0.34	0.000	0.11–0.40
No	0.45	0.97	0.411	0.07–3.00
Not sure	Ref			

Note: *SE* standard error; *OR* odds ratio; *CI* confidence interval

## Discussion

According to our survey, 78.7% of our participants said they would be willing to get vaccinated or would agree to vaccinate for who they were cared for, while 21.3% would refuse the vaccine. Most of them (71.3%) refused the vaccine for concerning its safety. In terms of preferences, participants would prefer a free vaccination, which was developed and funded by Chinese government. Participants would also prefer to be vaccinated in hospitals. Among those who intended to get vaccinated, factors influencing their vaccination acceptance were high vaccine coverage amongst community residents or their relatives and friends, the possibility of pandemic resurgence and the perceptions of vaccines as a solution to the pandemic.

The high acceptance of the COVID-19 vaccine amongst participants implied that their need for vaccines was evident. Since the COVID-19 outbreak, the Non-pharmaceutical interventions disrupted work and daily life across the population. As reported in the study, 77.7% of our participants agreed their daily routine was influenced at a fair level or above. As China recovered from the pandemic after the lockdown was lifted, only 13.0% of our participants considered that they had a high risk of being infected. Their reduced self-perceived risk of COVID-19 infection might lead to less compliance with restrictions measures (e.g., wearing masks) [23]. Given that therapeutics for COVID-19 have yet to be discovered, and the public’s adherence to restriction measures was weaken, a vaccine was needed to assure that normal life would continue and to prevent further waves of infection [13]. Chinese scientists have ramped up together to develop vaccines for COVID-19 at the earliest stage of the pandemic [24, 25]. Our research showed preference of Chinese state-developed vaccine. This contradicted other research findings where imported vaccines were more popular [26]. However, there is no indication of distrust towards any non-Chinese state-developed vaccines. There were several contributors for people’s preference: firstly, drastic measures such as national lockdown and its successful outcome had gained huge confidence for the Chinese government from its people; as a result, people trusted the government would push for an effective vaccine however difficult. Secondly, as Chinese government responded promptly to the pandemic and it was under controlled after national lockdown, there was more information gathered and more time and resources for Chinese scientists to develop a vaccine. Finally, daily update on new cases, as well as development of vaccines were reported everyday on special national news programme. Information is made available in all traditional media: newspaper, TV and radio as well as online social media platform: WeChat news, Tencent News, Weibo et al.

However, translating the willingness for vaccination into actual action is essential, yet complicated. Over the last decade, trust in vaccines has been increasingly challenged by vaccine scandals, such as the Hepatitis B vaccine crisis of 2014 [16, 27], and anti-vaccination sentiments [28]. In line with reduced trust, the vaccination dropped [29]. 21.3% of the participants said they would reject the vaccination, and the uncertainty about vaccine safety was our participants’ main reason to refuse the vaccine. They might worry that the medicine they or their family members already took would interact with the vaccine, as the current knowledge about this newly-launched vaccine was scarce and the proof of its immunogenicity and safety was short-term at present [30]. In this situation, providing timely and transparent information would help to reduce distrust in vaccination and convincing doubters to become vaccinated amongst this group. Furthermore, continued investigations into the efficacy and safety of the vaccine were required. Adverse events in ongoing research should be disseminated to the public with clear interpretation, or else negativity would become rumors that compounded vaccine hesitancy. After all, 63.9% in our study stated that negative news would reduce their possibility of receiving vaccination.

Our study revealed the vaccine coverage of community residents or friends and relatives around our participants was one of the factors that influenced the vaccination utility. An individual follows a morally neutral perception of what the majority of individual are doing is called descriptive social norms. According to previous study, social norms could either support or hinder vaccination goals [31] and norms-base strategies might increase vaccination intention [32]. Public health messaging should update vaccination coverage to encourage psychiatric patients to receive vaccination. Our study also found that participants’ vaccination intention related to the possible resurgence of the COVID-19 pandemic and the thought that the vaccine is the most powerful weapon with which to combat the virus. Under the current circumstance, it seemed likely that COVID-19 would co-exist with us and become a flu-like seasonal disease [30]. As a result, the number of newly infected COVID-19 cases should be released timely to remind the participants that the pandemic was under control but far from over, getting infected was possible. The importance of vaccine as an effective measure to prevent themselves from serious illness and hospitalization, and control the virus should be stressed in tailored communication. Our results showed that the influencing factors underlying vaccination acceptance between psychiatric patients and the general public, might be different [33]. Utilizing evidence-based communication strategies is essential, if we are to achieve an adequate vaccination rate.

In our study, we focused on psychiatric patients, aiming to evaluate their attitude towards COVID-19 vaccination, which provided information for the acceptance of COVID-19 vaccination by this vulnerable group. By considering the vaccine distrust and dividing subjects based on vaccine acceptance, our study revealed influencing factors that contributed to the participants’ decision-making process with regards to vaccination, such as the vaccine coverage of their surrounding people. The investigation of barriers and preference of vaccination was useful when government needed to generate effective immunization strategies for this group. However, this study has several limitations. First, a modest sample size of 408 participants was enrolled from the same hospital. Therefore, our findings may not adequately reflect trends seen throughout the whole targeted population. Second, the study conducted before a COVID-19 vaccine was available to the public. The results may differ when a vaccine was ready for the immunization program. Finally, family caregivers were recruited for those patients who were incapable of making medical decisions, whose opinion might not be the same as the patients they looked after. Future studies should enroll a larger sample size from different hospitals after a vaccine approved to be used. More research could be done to investigate family caregivers’ role in supporting the psychiatric patients’ acceptance of COVID-19 vaccination.

## Conclusion

To conclude, this study reflected a high level of acceptance of COVID-19 vaccination amongst our psychiatric patients. For those who would decline vaccination, vaccine safety was their main concern. It is important to remind the public that vaccination plays a crucial part in containing COVID-19. Timely information including new COVID-19 cases in the regions and up-to-date vaccinated number of people amongst community can help to increase vaccination uptake. Improving health literacy of vaccine safety is crucial to ease concerns amongst this vulnerable group.

## Supplementary Information

**Additional file 1: Table S1.** Questionnaire

**Additional file 2: Table S2.** Comparison of baseline characteristics between the vaccine-accept group and vaccine-refuse group.

## Data Availability

The datasets used and analyzed during the current study are available from the corresponding author on reasonable request.
