# Supplementary material for: The attitudes of psychiatric patients towards COVID-19 vaccination in China: a cross-sectional study
Source: BMC Psychiatry. 2021 Sep 29;21:475. doi: 10.1186/s12888-021-03484-9 (PMC8479711; doi:10.1186/s12888-021-03484-9)
Supplement: Supplementary file 1 — Additional file 1: Table S1. Questionnaire [file 12888_2021_3484_MOESM1_ESM.docx]

Table S1. Questionnaire

| Consent for participation |
| --- |
| ◎Agree |
| ◎Disagree |
| 1. Identity |
| ◎I have been diagnosed with a mental illness |
| ◎I am a family member of mental illness |
| 1. What is your diagnosis? |
| ◎Schizophrenia |
| ◎Bipolar Disorder |
| ◎Major Depression Disorder |
| ◎Generalized Anxiety Disorder |
| ◎Others |
| 1. What is your sex? |
| ◎Male |
| ◎Female |
| 1. How old are you? |
| ◎18-44 |
| ◎45-59 |
| ◎60 and above |
| 1. What is your marriage status? |
| ◎Married |
| ◎Unmarried |
| ◎Others (divorced, widowed) |
| 1. What is your highest level of education? |
| ◎Primary school and below |
| ◎Middle or high school |
| ◎Bachelor and above |
| 1. What is your working status? |
| ◎Full-time employed |
| ◎Part-time employed |
| ◎Students |
| ◎Retired |
| ◎Unemployed |
| 1. What region do you live? |
| ◎Urban |
| ◎Rural |
| 1. Who do you live with? |
| ◎Independent or with partners |
| ◎With parents |
| ◎With children |
| ◎With parents and children |
| 1. What is your personal annual income? |
| ◎Less or equal to 60,000 (￥) |
| ◎Over 60,000 (￥) |
| 1. how do you think of your health status? |
| ◎Good |
| ◎Fair |
| ◎Poor |
| 1. How do you perceived your risk of getting COVID-19 infected |
| ◎High or very high |
| ◎Fair |
| ◎Low or very low |
| 1. Did the pandemic affect your daily life or work? |
| ◎Large or very large |
| ◎Fair |
| ◎Small or very small |
| 1. Did the pandemic affect your income? |
| ◎Large or very large |
| ◎Fair |
| ◎Small or very small |
| 1. Did you receive Flu vaccination in past seasons? |
| ◎Yes |
| ◎No |
| 1. Do you know anyone who might have been vaccinated against COVID-19? |
| ◎Yes |
| ◎No |
| 1. Where do you get information about COVID-19 vaccines from most often? |
| ◎Mass media |
| ◎Social media |
| ◎Authorities |
| ◎Others |
| 1. Which best describe your understanding about COVID-19 vaccines? |
| ◎Very clear |
| ◎Roughly clear |
| ◎Not clear at all |
| 1. What concern you the most about COVID-19 vaccines? |
| ◎Safety |
| ◎Effectiveness |
| ◎Protection duration |
| 1. Would you like to be vaccinated if COVID-19 vaccines become available? |
| ◎Yes |
| ◎No |
| 20a. What is the reason for rejecting vaccination? |
| ◎Worry about the cost |
| ◎Worry about the safety |
| ◎Worry about the mutation |
| 1. Will you encourage others to get vaccinated? |
| ◎Yes |
| ◎No |
| 1. Which of the following people's suggestions would increase your likelihood of getting vaccinated? |
| ◎Friends |
| ◎Family |
| ◎Authority or Doctors |
| 1. You are more likely to have vaccines if there is high vaccine coverage amongst community residents or relatives and friends. |
| ◎Yes |
| ◎No |
| 1. You are more likely to have vaccines if the pandemic returns. |
| ◎Yes |
| ◎No |
| 1. You are more likely to have vaccines in order to protect children or the elderly in your family. |
| ◎Yes |
| ◎No |
| 1. Which place do you think is most suitable for vaccination? |
| ◎Community health centre |
| ◎Health screen centre |
| ◎Hospitals |
| 1. How do you think vaccines should be charged? |
| ◎Free |
| ◎Partially paid by individual |
| ◎All paid by individual |
| 1. Which vaccine would you prefer to be vaccinated? |
| ◎Imported |
| ◎Chinese vaccines |
| 1. You are less likely to have vaccines if negative news reported against COVID-19 vaccines. |
| ◎Yes |
| ◎No |
| 1. Do you think the COVID-19 vaccine is crucial to control the pandemic? |
| ◎Yes |
| ◎No |
| ◎Not sure |
| 1. Do you have any concerns over COVID-19 and its vaccination? Please fill in the blank below. |
| ◎ |
