# Supplementary material for: The attitudes of psychiatric patients towards COVID-19 vaccination in China: a cross-sectional study
Source: BMC Psychiatry. 2021 Sep 29;21:475. doi: 10.1186/s12888-021-03484-9 (PMC8479711; doi:10.1186/s12888-021-03484-9)
Supplement: Supplementary file 2 — Additional file 2: Table S2. Comparison of baseline characteristics between the vaccine-accept group and vaccine-refuse group. [file 12888_2021_3484_MOESM2_ESM.docx]

Table S2. Comparison of baseline characteristics between the vaccine-accept group and vaccine-refuse group.

| Items | | The willingness to get vaccinated | | | Chi-  square | *p*- value* |
| --- | --- | --- | --- | --- | --- | --- |
|  |  | Yes | No | |  |  |
| Identity | |  |  | |  |  |
|  | Family caregiver | 143 | 36 | | 6.140 | 0.293 |
|  | Patient | 178 | 51 | |  |  |
| Sex | | | | |  |  |
|  | Male | 127 | | 23 | 5.074 | 0.025 |
|  | Female | 194 | | 64 |  |  |
| Age group | | | | |  |  |
|  | 18-44 | 205 | | 66 | 6.990 | 0.030 |
|  | 45-59 | 97 | | 14 |  |  |
|  | 60 and above | 19 | | 7 |  |  |
| Marriage status | | | | |  |  |
|  | Married | 179 | | 49 | 0.009 | 0.996 |
|  | Unmarried | 127 | | 34 |  |  |
|  | Others (divorced, widowed) | 15 | | 4 |  |  |
| Highest level of education | | | | |  |  |
|  | Primary school and below | 6 | | 1 | 3.162 | 0.206 |
|  | Middle or high school | 131 | | 27 |  |  |
|  | Bachelor and above | 184 | | 59 |  |  |
| Working status | | | | |  |  |
|  | Full-time employed | 164 | | 48 | 4.042 | 0.400 |
|  | Part-time employed | 40 | | 15 |  |  |
|  | Students | 56 | | 9 |  |  |
|  | Retired | 38 | | 8 |  |  |
|  | Unemployed | 23 | | 7 |  |  |
| Region | | | | |  |  |
|  | Urban | 258 | | 74 | 0.991 | 0.355 |
|  | Rural | 63 | | 13 |  |  |
| Living status | | | | |  |  |
|  | Independent or with partners | 88 | | 21 | 5.044 | 0.169 |
|  | With parents | 94 | | 31 |  |  |
|  | With children | 103 | | 20 |  |  |
|  | With parents and children | 36 | | 15 |  |  |
| Annual personal income | | | | |  |  |
|  | Less or equal to 60,000 (￥) | 85 | | 24 | 0.043 | 0.891 |
|  | Over 60,000 (￥) | 236 | | 63 |  |  |
| Health status | | | | |  |  |
|  | Good | 139 | | 42 | 1.115 | 0.573 |
|  | Fair | 164 | | 39 |  |  |
|  | Poor | 18 | | 6 |  |  |
| Perceived risk of infection | | | | |  |  |
|  | High or very high | 45 | | 8 | 7.472 | 0.024 |
|  | Fair | 144 | | 29 |  |  |
|  | Low or very low | 132 | | 50 |  |  |
| Pandemic impact on daily life or work | | | | |  |  |
|  | Large or very large | 113 | | 24 | 3.238 | 0.198 |
|  | Fair | 142 | | 38 |  |  |
|  | Small or very small | 66 | | 25 |  |  |
| Pandemic impact on income | | | | |  |  |
|  | Large or very large | 73 | | 14 | 2.196 | 0.334 |
|  | Fair | 129 | | 35 |  |  |
|  | Small or very small | 119 | | 38 |  |  |
| Received Flu vaccination in past seasons | | | | |  |  |
|  | Yes | 75 | | 13 | 2.870 | 0.106 |
|  | No | 246 | | 74 |  |  |
| Do you know anyone who might have been vaccinated against COVID-19? | | | | |  |  |
|  | Yes | 281 | | 73 | 0.786 | 0.376 |
|  | No | 40 | | 14 |  |  |
| How do you perceived your risk of getting COVID-19 infected? | | | | |  |  |
|  | Totally clear | 38 | | 9 | 3.893 | 0.143 |
|  | Roughly clear | 186 | | 42 |  |  |
|  | Not clear at all | 97 | | 36 |  |  |
| Will you encourage others to get vaccinated? | | | | |  |  |
|  | Yes | 256 | | 7 | 153.623 | 0 |
|  | No | 65 | | 80 |  |  |
| You are more likely to have vaccines if there is high vaccine coverage amongst community residents or relatives and friends. | | | | |  |  |
|  | Yes | 306 | | 54 | 72.935 | 0 |
|  | No | 15 | | 33 |  |  |
| You are more likely to have vaccines if the pandemic returns. | | | | |  |  |
|  | Yes | 312 | | 52 | 99.651 | 0 |
|  | No | 9 | | 35 |  |  |
| You are more likely to have vaccines in order to protect children or the elderly in your family. | | | | |  |  |
|  | Yes | 308 | | 59 | 59.938 | 0 |
|  | No | 13 | | 28 |  |  |
| You are less likely to have vaccines if negative news reported against COVID-19 vaccines. | | | | |  |  |
|  | Yes | 193 | | 67 | 8.444 | 0.004 |
|  | No | 128 | | 20 |  |  |
| Do you think the COVID-19 vaccine is crucial to control the pandemic? | | | | |  |  |
|  | Yes | 283 | | 42 | 67.210 | 0 |
|  | No | 4 | | 5 |  |  |
|  | Not sure | 34 | | 40 |  |  |

*: *p-*values <0.05
